# Spondylodiscitis as osteoarticular involvement in brucellosis

**DOI:** 10.1590/0037-8682-0461-2022

**Published:** 2023-01-23

**Authors:** Mehmet Kürşat Karadağ, Handan Alay, Bahar Yılmaz Çankaya

**Affiliations:** 1 Ataturk University, Faculty of Medicine, Department of Neurosurgery, Erzurum, Turkey.; 2 Ataturk University, Faculty of Medicine, Department of Infectious Diseases and Clinical Microbiology, Erzurum, Turkey.; 3 Ataturk University, Faculty of Medicine, Department of Radiology, Erzurum, Turkey.

**FIGURE 1:**
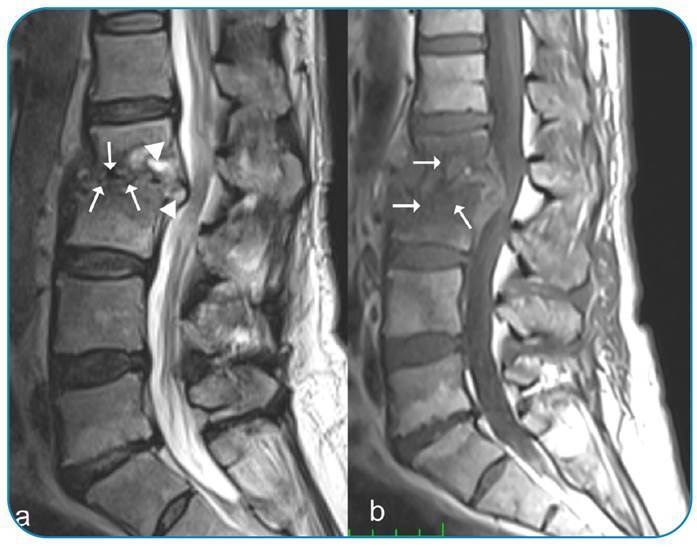
Pretreatment MRI scan. **(a)** The sagittal T2-weighted image without fat suppression shows erosions (arrow), effusion, and hyperintense signals in the disc space and epidural area (arrowheads) in the lower L2 vertebral and upper L3 vertebral endplates. **(b)** The sagittal T1-weighted image without fat suppression shows marked signal losses (arrow) in the vertebral bodies and disc space.

**FIGURE 2:**
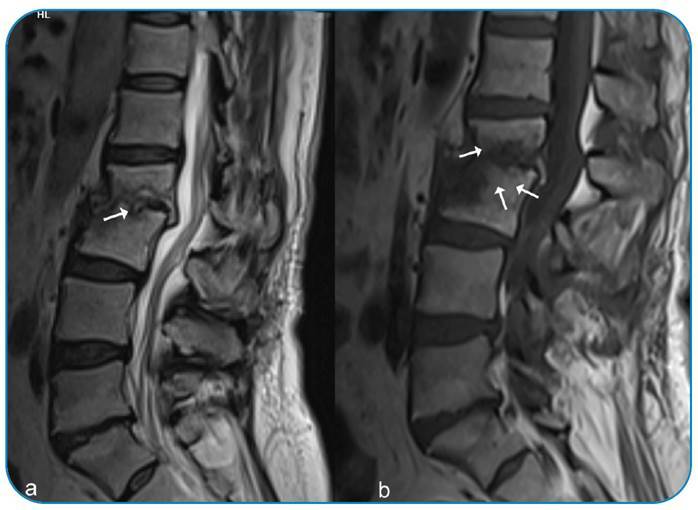
Posttreatment MRI scan. **(a)** The sagittal T2-weighted image without fat suppression shows persisting erosions in the vertebral endplates (arrow) but resolution of the hyperintense signals in the disc space and epidural area. **(b)** The sagittal T1-weighted image without fat suppression shows decreased signal losses (arrows) in the vertebral bodies and disc space.

A 54-year-old woman presented with weight loss of approximately 30 kg, severe lumbar pain, pain-related inability to walk, and limitation in physical activities for 1 year. She had a history of consuming unpasteurized milk and milk products despite living in the city center region. Physical examination showed restricted vertebral movements. Lumbar tomography and magnetic resonance imaging (MRI) were performed (Figure 1). The patient was referred for brain surgery. Pulmonary computed tomography showed no abnormalities. Blood tests showed a white blood cell count of 6.07 × 10^3^/µL, a C-reactive protein (CRP) level of 14.78 mg/L, and an erythrocyte sedimentation rate of 83 mm/h. The tuberculin skin test result was 0 mm. Her Wright agglutination and brucella IgM and IgG test results were 1/1280, 1.55, and 2.98 (cutoff: 0.9-1.1), respectively. The patient was started on doxycycline 2 × 100 mg, rifampicin 1 × 600 mg, and streptomycin 1 g/day for 21 days. In week 2 of treatment, the sedimentation rate and CRP level decreased to 23 mm/h and 3.7 mg/L, respectively. Pain was alleviated, and movement restriction was resolved. Figure 2 shows the MRI scan acquired after 5 months of treatment.

Brucellosis is a disease affecting the musculoskeletal system. Clinically, it can mimic several diseases^1^. Lumbar pain is the main symptom of spondylodiscitis but non-specific. This results in delayed diagnosis and treatment^2^. Patients with spinal brucellosis with lumbar pain and sciatic radiculopathy may be misdiagnosed with intervertebral disc disease and undergo surgery^3^. Serological screening tests are essential in such patients in endemic regions.
